# Activation of the Mitochondrial Apoptotic Signaling Platform during Rubella Virus Infection

**DOI:** 10.3390/v7122928

**Published:** 2015-11-26

**Authors:** Claudia Claus, Lena Manssen, Denise Hübner, Sarah Roßmark, Viktoria Bothe, Alice Petzold, Claudia Große, Mareen Reins, Annette Mankertz, Teryl K. Frey, Uwe G. Liebert

**Affiliations:** 1 Institute of Virology, University of Leipzig, 04103 Leipzig, Germany; lenamanssen@yahoo.de (L.M.); denise.hübner@medizin.uni-leipzig.de (D.H.); sarah.rossmark@medizin.uni-leipzig.de (S.R.); alice_petzold@web.de (A.P.); claudia.268@web.de (C.G.); mareen.reins@medizin.uni-leipzig.de (M.R.); liebert@medizin.uni-leipzig.de (U.G.L.); 2 Division of Clinical Pharmacology, Ludwig-Maximilian University Munich, 80336 Munich, Germany; viktoria.bothe@gmx.de; 3 WHO European Regional Reference Laboratory for Measles and Rubella, Robert Koch-Institute, 13353 Berlin, Germany; MankertzA@rki.de; 4 Department of Biology, Georgia State University, Atlanta, GA 30303, USA; zfrey@bellsouth.net

**Keywords:** mPTP, p53, AIF, cyclophilin family, CypA, Cyp40, cytochrome c, NIM811, PFTμ

## Abstract

Mitochondria- as well as p53-based signaling pathways are central for the execution of the intrinsic apoptotic cascade. Their contribution to rubella virus (RV)-induced apoptosis was addressed through time-specific evaluation of characteristic parameters such as permeabilization of the mitochondrial membrane and subsequent release of the pro-apoptotic proteins apoptosis-inducing factor (AIF) and cytochrome c from mitochondria. Additionally, expression and localization pattern of p53 and selected members of the multifunctional and stress-inducible cyclophilin family were examined. The application of pifithrin μ as an inhibitor of p53 shuttling to mitochondria reduced RV-induced cell death to an extent similar to that of the broad spectrum caspase inhibitor z-VAD-fmk (benzyloxycarbonyl-V-A-D-(OMe)-fmk). However, RV progeny generation was not altered. This indicates that, despite an increased survival rate of its cellular host, induction of apoptosis neither supports nor restricts RV replication. Moreover, some of the examined apoptotic markers were affected in a strain-specific manner and differed between the cell culture-adapted strains: Therien and the HPV77 vaccine on the one hand, and a clinical isolate on the other. In summary, the results presented indicate that the transcription-independent mitochondrial p53 program contributes to RV-induced apoptosis.

## 1. Introduction

Rubella virus (RV) as the only member of the *Rubivirus* genus in the family *Togaviridae* causes a mild childhood disease, but acts as an extremely efficient teratogen when infection occurs during the first trimester of pregnancy. The virus particle consists of an envelope with the two glycoproteins E1 and E2 and the nucleocapsid, which comprises a protein coat composed of the capsid (C) and the single-stranded positive-sense RNA genome [1].

RV-induced apoptosis occurs in a complex, multi-step and rather cell type-specific manner [2]. Moreover, precise mechanisms remain to be resolved as reports on the involvement of p53-independent [3,4] as well as p53-dependent mechanisms [5] during RV-induced cell death are conflicting. Additionally, prolonged survival of RV-infected cells is ensured by the induction of the phosphatidylinositol 3′-kinase (PI3K)/AKT survival pathway [6] and by anti-apoptotic activities of the viral C protein [7,8]. These viral infection-promoting activities of the C protein involve its localization to mitochondria and its interaction with the pro-apoptotic protein B-cell lymphoma-2 (Bcl-2)-associated X protein (Bax) and the mitochondrial matrix protein p32 (gC1qR), [7,9]. The p32 protein is required for viral replication [10] and for transport of mitochondria to viral replication complexes [11]. In addition to its interaction with mitochondrial proteins, RV infection has an impact on mitochondrial bioenergetic function [11,12].

Due to the interdependency of apoptotic and metabolic pathways [13], the mitochondria-based signaling platform might contribute to RV-associated programmed cell death. The intrinsic mitochondrial apoptotic pathway can be induced by cytotoxic stress during ongoing viral replication and is usually accompanied by permeabilization of the inner (IMM) and/or outer (OMM) mitochondrial membrane. Mitochondrial permeabilization is characterized by formation of “death decision pores”, such as ceramide lipid pores; the mitochondrial apoptosis-induced channel (MAC) formed in response to OMM permeabilization (MOMP); and the relatively large mitochondrial permeability transition pore (mPTP), which originates at the IMM [14]. MOMP and subsequently MAC formation can result from oligomerization of Bcl-2 family members such as Bax and Bcl-2 homologous antagonist killer (Bak). Through the formation of these death decision pores, mitochondrial function is lost and the apoptotic cascade is further fueled, as metabolites, small ions and apoptogenic factors such as cytochrome c (Cytc), Smac/Diablo, apoptosis-inducing factor (AIF) and/or endonuclease G (Endo G) are released. The coordination of these processes involves the tumor-suppressor protein p53, which executes its function through both a transcription-dependent (nuclear) and transcription-independent (mitochondrial) pathway. The former influences the mRNA level of pro- and anti-apoptotic factors and the latter involves direct regulation of protein functions at mitochondria, e.g., activation of the pro-apoptotic Bax and Bak proteins [15]. Additionally, p53 might also interact directly with mitochondria and induce MOMP by itself [16].

The focus of the present study is set at disclosing the contribution of mitochondria (namely the mPTP and translocation of mitochondrial pro-apoptotic proteins), p53, and selected members of the stress-inducible cyclophilin family to RV-induced apoptosis. The multifunctional cyclophilins as proteins of the peptidyl-prolyl cis-trans isomerase (PPIase) family are highly conserved molecular chaperons that support protein folding and isomerization and thus participate in the cellular stress response [17]. To study the contribution of apoptosis-promoting parameters to RV-associated cellular aberrations, selected pharmacological compounds were applied to RV-infected cells. Presented data point to a contribution of mitochondrial translocation of p53, partial opening of the mPTP and nuclear shuttling of AIF and cyclophilin 40 (Cyp40) to RV-induced apoptosis, which occurs at least partly in a strain-specific manner.

## 2. Results

### 2.1. Effect of Pharmacological Inhibitors of Apoptotic Signaling Pathways on Rubella Virus-Induced Cell Death

**Figure 1:**
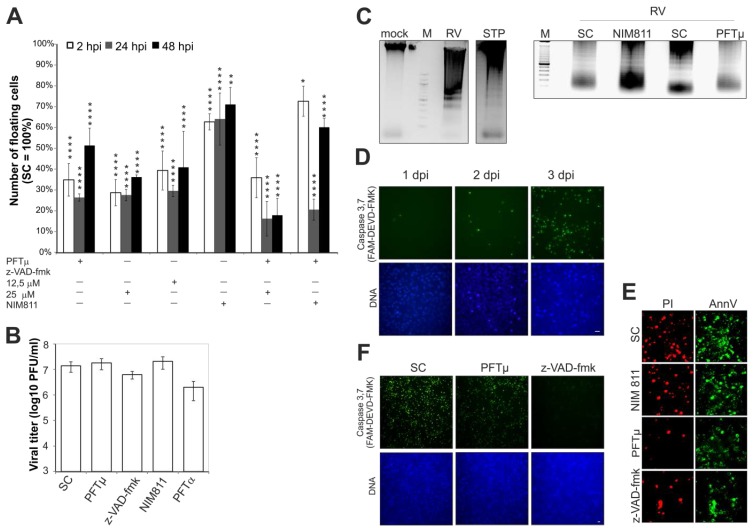
Analysis of selected pharmacological inhibitors (z-VAD-fmk (12.5 μM if not otherwise indicated), PFTμ and α (12.5 μM), and NIM811 (2 μM)) with respect to RV replication and cytopathic effect (CPE) induction. (**A**) Effect of different application time points on the presence of floaters in the supernatant of RV-infected Vero cells at 3 dpi. The respective SC (DMSO)-treated sample was set at 100%; (**B**) RV titer was determined by plaque assay for supernatants collected at 3 dpi after drug application at the time point with maximal efficacy (NIM811 at 2 hpi, z-VAD-FMK and PFTμ at 24 hpi). PFT α was added at 2 hpi. In comparison to the SC, changes in viral titer were not significant; (**C**) Characterization of the presence of an apoptotic laddering in the floater population collected from the supernatant of the SC- and NIM811 (2 hpi)- and PFTμ (24 hpi)-treated and RV-infected Vero cells. Floaters were collected at 3 dpi and then counted before the same number of floaters (1 × 10^6^) was subjected to DNA extraction and analysis by agarose gel electrophoresis. As a control, mock- and staurosporine (5 μM)-treated Vero cells are shown in comparison to the floater population (3 dpi) of RV-infected cells. M, molecular size marker; (**D**) Time course analysis (1, 2, 3 dpi) of the activation of caspase 3 and 7 in RV-infected Vero cells; (**E**) Analysis of necrotic (Propidium iodide (PI)-positive) and early (annexin V-enhanced green fluorescent protein (EGFP)-positive) and late-apoptotic events (annexin V-enhanced green fluorescent protein (EGFP) and PI-positive) by annexin V-EGFP and PI staining by microscopic analysis of living cells; (**F**) Determination of caspase 3 and 7 activity after application of PFTμ and NIM811 in comparison to the SC. SC, solvent control; scale bar, 20 μm. * *p* < 0.05, ** *p* < 0.01, *** *p* < 0.001, **** *p* < 0.0001.

Three specific pharmacological inhibitors were used to explore RV-induced apoptotic pathways. The pan caspase inhibitor z-VAD-fmk as an already-described inhibitor of RV-induced apoptosis [7,18] was applied as a positive control to assess the effectiveness of *N*-methyl-4-isoleucine cyclosporine (NIM811) and pifithrin (PFT) μ. The cyclosporine A analog NIM811 is a non-immunosuppressive cyclophilin inhibitor. PFTμ is an inhibitor of the mitochondrial translocation of p53, which reduces the binding affinity of p53 to B cell lymphoma-extra large (Bcl-xL) and acts upstream of Bax [19]. Time course experiments were performed to determine the optimal time point for addition of the respective inhibitor. As an indicator for RV-induced cell death, the number of detached cells (the floaters) in the supernatant can be determined [18]. Hence compound effectiveness was assessed at 72 h post-infection (hpi) through harvesting and counting the number of floaters. Figure 1A shows the effect of z-VAD-fmk at 12.5 and 25 μM, PFTμ at 12.5 μM and NIM811 at 2 μM (applied at 2, 24 and 48 hpi) on the floater population. In comparison to the respective solvent control (SC, set at 100%), the number of floaters was significantly reduced by all three compounds (Figure 1A), but at varying degrees, which was also dependent on the application time point. NIM811 was the least effective compound, while the effectiveness of PFTμ was comparable to z-VAD-fmk. The greatest level of reduction in the floater population was reached after application of PFTμ (27% ± 2% of the SC) and z-VAD-fmk (30% ± 3% and 28% ± 3% of the SC for 12.5 μM and 25 μM, respectively) at 24 hpi.

For subsequent experiments z-VAD-fmk was used at 12.5 μM such that the lowest possible effective concentration was used for its application at 24 hpi. While PFTμ and z-VAD-fmk were both applied at 24 hpi, 2 hpi was the selected application time point of NIM811, as this time point was slightly more effective than the one at 24 hpi. Figure 1B indicates that viral titer was not affected by application of these inhibitors, which otherwise could also result in a reduction of RV-associated cellular aberrations. The pharmacological compound PFTα blocks p53-mediated transcription [20]. PFTα was thus applied to RV-infected Vero cells to test the second branch of p53 activity, which is based on its function as a transcription factor. However, in contrast to PFTμ, PFTα had a noticeable negative effect on viral progeny generation (Figure 1B), hence it was excluded from subsequent experiments.

The analysis of the mode of cell death after application of the pharmacological compounds began with the verification of the presence of DNA fragmentation within the floater population as a hallmark of apoptosis in RV-infected cells. Application of z-VAD-fmk was excluded from the analysis as it is a well-characterized inhibitor of apoptosis. Figure 1C shows a clear laddering of oligonucleosomal fragments and indicates that floaters possess DNA fragmentation irrespective to the application of pharmacological compounds.

Figure 1D indicates that with 72 h post-infection only at late time points of infection the activity of caspase 3 and 7 as executioner caspases was detectable at a significant level in RV-infected cells. Hence, this time point was used for studying the effect of NIM811, z-VAD-fmk and PFTμ on RV-induced apoptotic signaling pathways. Figure 1E shows conventional annexin V-enhanced green fluorescent protein (EGFP)/propidium iodide (PI) staining and indicates that in comparison to the SC, the number of PI- and annexin V-EGFP-positive cells was reduced after application of z-VAD-fmk and to a lesser extent after treatment with PFTμ, but not after addition of NIM811. To extend this observation, activity of caspase 3 and 7 was determined after application of PFTμ and z-VAD-fmk. Figure 1F depicts the reduction in active caspase 3 and 7 which as expected was profound after application of z-VAD-fmk. In comparison to the SC, PFTμ also reduces the amount of active caspase 3 and 7, but not as effective as z-VAD-fmk (Figure 1F). Taken together, PFTμ appears to target one of the key players during RV-induced cell death.

### 2.2. Rubella Virus Infection Results in Partial Opening of the Mitochondrial Permeability Transition Pore

**Figure 2:**
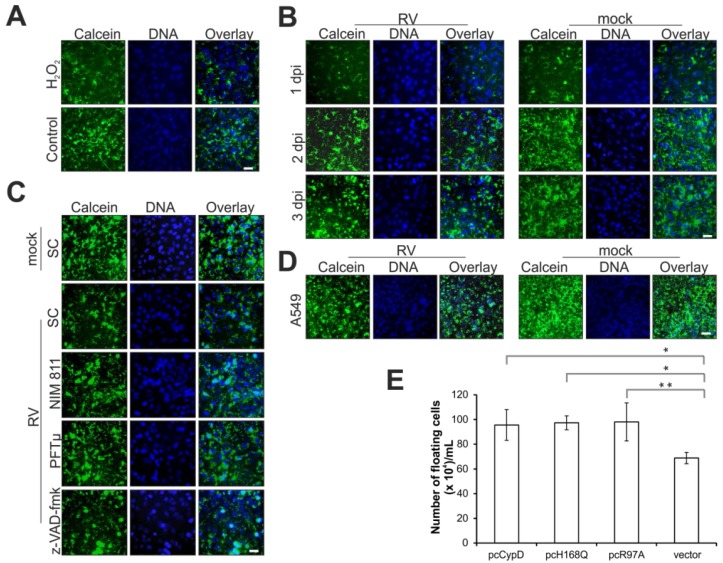
Effect of RV replication (in the presence and absence of indicated pharmacological inhibitors) on the mPTP. (**A**), (**B**), (**C**), (**D**) mPTP opening was assessed through application of calcein-AM. Cytosolic calcein was quenched with CoCl_2_. Calcein signal is shown in green, nuclear DNA in blue, cyan colour results from the overlay of both imgaes. (**A**) mPTP opening was induced by overnight incubation of Vero cells with 0.003% H_2_O_2_; (**B**) Time course analysis (1, 2, 3 dpi) of mPTP opening under RV infection; (**C**) Three days after infection with RV, the effect of the pharmacological inhibitors NIM811 (2 μM) at 2 hpi, z-VAD-fmk and PFTμ (both at 12.5 μM) at 24 hpi on the mPTP opening was assessed in comparison to the SC; (**D**) Analysis of mPTP opening in RV- and mock-infected A549 cells at 3 dpi; (**E**) RV-infected Vero cells were transfected at 2 hpi with plasmids encoding native CypD protein and mutants of the PPIase domain (H168Q and R97A). Number of floaters was determined at 3 dpi in comparison to the vector control (pcDNA3). SC, solvent control; scale bar, 20 μm. * *p* < 0.05, ** *p* < 0.01, *** *p* < 0.001, **** *p* < 0.0001.

In this study, the opening of the mPTP was examined through application of the nonfluorescent, cell-permeant dye calcein-acetoxymethyl (AM), which is converted into the green-fluorescent dye calcein after acetoxymethyl ester hydrolysis by intracellular esterases. Figure 2A shows that overnight incubation of Vero cells with 0.003% H_2_O_2_ induces massive loss of calcein from mitochondria due to pore opening. Thereafter a time course experiment was performed to identify mPTP opening during RV infection. Figure 2B indicates that, with 72 h post-infection as a rather late time point of infection, a notable portion of cells was positive for mPTP opening.

Whereas the addition of z-VAD-fmk had no detectable influence on opening of the mPTP, the application of NIM811 and PFTμ appears to reduce its opening as indicated by an increase in the calcein signal (Figure 2C). To extend this data to Vero cells, Figure 2D highlights that after infection of A549 with RV, a loss of calcein from mitochondria was also observable at 3 dpi.

The mitochondrial cyclophilin D (CypD) is a discussed regulatory component of the mPTP [21,22,23]. Taking this into account, the CPE induction by RV was assessed after overexpression of CypD and its PPIase mutants H168Q and R97A lacking the catalytic function as a peptidyl-prolyl isomerase. Figure 2E shows, that at 3 dpi and in comparison to the vector control (pcDNA3), overexpression of CypD and its PPIase mutants H168Q and R97A increases the number of floaters present in the supernatant of RV-infected Vero cells. In summary, at late time points of infection, RV induces a partial opening of the mPTP.

### 2.3. Rubella Virus Induces Nuclear Translocation of the Pro-Apoptotic Mitochondrial Protein Apoptosis-Inducing Factor (AIF)

**Figure 3:**
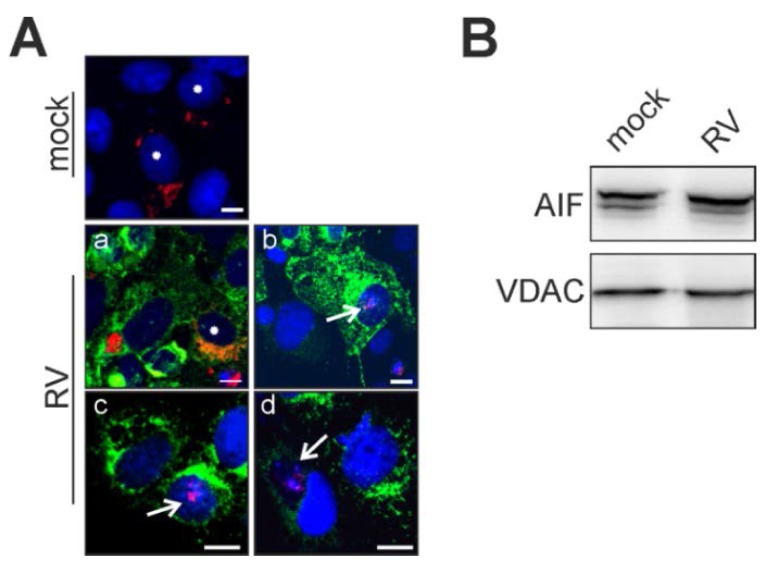
Release of the mitochondrial death-effector protein AIF. Vero cells overexpressing AIF-TagRFP were applied at 2 dpi (**A**) for determination of RV-induced translocation of AIF-TagRFP (shown in red) from mitochondria to the nucleus. Nuclear DNA is shown in blue, the viral E1 protein is shown in green. Asterisks (mock and Aa) indicate physiological localization of AIF to mitochondria. Arrows (Ab,c and d) highlight cells with nuclear association of AIF; (**B**) 30 μg of mitochondrial fractions obtained at 3 dpi from mock- and RV-infected Vero cells were subjected to Western blot analysis with anti-AIF antibody. As a loading control, anti-VDAC antibody was applied. Scale bar, 10 μm.

After assessment of the mPTP opening, intracellular distribution of AIF was documented after its overexpression. At 2 hpi, mock- and RV-infected Vero cells were transfected with a plasmid encoding AIF-TagRFP (red fluorescent protein). At 48 h post-infection and post-transfection, distribution of AIF-TagRFP was analyzed. Upon RV infection, AIF-TagRFP can be found in mitochondria, as expected, and as it was oberserved in mock-infected cells (Figure 3A). However, in contrast to the mock infection, AIF-TagRFP can also be found in the nucleus and especially in areas of condensed chromatin (Figure 3Aa–d). In some cases, nuclear counterstaining with Hoechst bisbenzamide was hardly traceable (Figure 3Ad). However, Western blot analysis with anti-AIF antibody revealed no detectable change in AIF concentration in the mitochondria-enriched fraction derived at 3 dpi from RV-infected Vero cells as compared to the one obtained from mock-infected cells (Figure 3B). This indicates that only a minor portion of mitochondrial AIF translocates towards the nucleus during RV infection.

### 2.4. Point of No Return: Release of the Apoptosis-Promoting Protein Cytochrome C from Mitochondria

**Figure 4:**
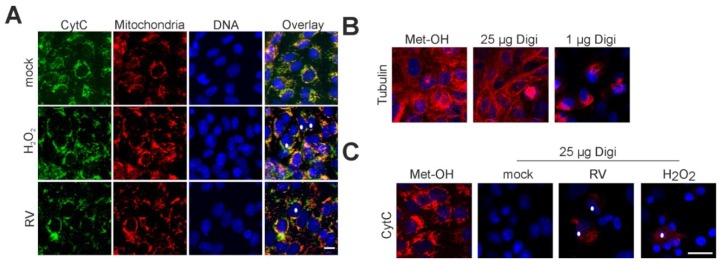
Localization of the mitochondrial death-effector protein cytochrome c under RV infection. (**A**) Immunofluorescence analysis of RV- and mock-infected Vero cells at 3 dpi with anti- CytC antibody (shown in green). As a counterstain, mitochondria were labelled with MitoTracker Red CMXRos (shown in red) before fixation. Nuclear DNA is shown in blue, yellow colour indicates overlap areas. As a positive control, overnight incubation with 0.003% H_2_O_2_ was applied; (**B**) Selective permeabilization of the plasma membrane with digitonin was tested for alpha-tubulin (shown in red) as a representative cytoplasmic protein; (**C**) Selective permeabilization with digitonin at 25 μg/mL was applied to mock- and RV-infected Vero cells. Immunofluorescence was performed with anti-CytC antibody (shown in red). Release of CytC was induced through overnight incubation with 0.003% H_2_O_2_. (**B**),(**C**) As a control, immunofluorescence analysis of mock-infected Vero cells was performed after methanol (Met-OH) permeabilization. Asterisks highlight representative cells with localization of CytC outside of mitochondria. Scale bar, 10 μm.

Immunofluorescence analysis of RV- and mock-infected cells was applied to validate CytC localization during RV infection. MitoTracker Red CMXRos (Thermo Fisher Scientific, Braunschweig, Gemany) was used as a mitochondrial counterstain. Figure 4A highlights the strict mitochondrial localization of CytC in mock-infected Vero cells.

On the contrary, overnight incubation with 0.003% H_2_O_2_ induces profound localization of CytC outside of mitochondria (Figure 4A). In the case of RV-infected Vero cells (3 dpi), a minor but distinct portion of cells appear to have lost CytC from their mitochondria (Figure 4A). To further validate this observation, selective permeabilization of the plasma membrane with digitonin was performed. First, the lowest possible concentration of digitonin was determined, which gives a detectable fluorescence signal for a cytoplasmic protein, in this case alpha-tubulin. As a positive control, Figure 4B,C illustrate the signal for the cytoplasmic protein alpha-tubulin and the mitochondrial protein CytC after 2% (*w*/*v*) paraformaldehyde (PFA) fixation and permeabilization with methanol. Figure 4B highlights that permeabilization with digitonin at 25 μg/mL after 2% (*w*/*v*) PFA fixation gives a sufficient signal for alpha-tubulin after application of its respective antibody. However, a remarkable signal loss occurs after application of digitonin at 1 μg/mL (Figure 4B). Hence, digitonin was applied at 25 μg/mL to verify CytC localization outside of mitochondria. As shown in Figure 4C, no CytC signal can be detected in mock-treated cells. However, overnight incubation with 0.003% H_2_O_2_ produces a distinct CytC signal in the cytoplasm, which was also detectable for RV-infected cells (3 dpi). In summary, single cells within the RV-infected population were positive for CytC-signals in the cytoplasm, outside of mitochondria.

### 2.5. Cellular Distribution of p53 Is Altered during Rubella Virus Infection

**Figure 5:**
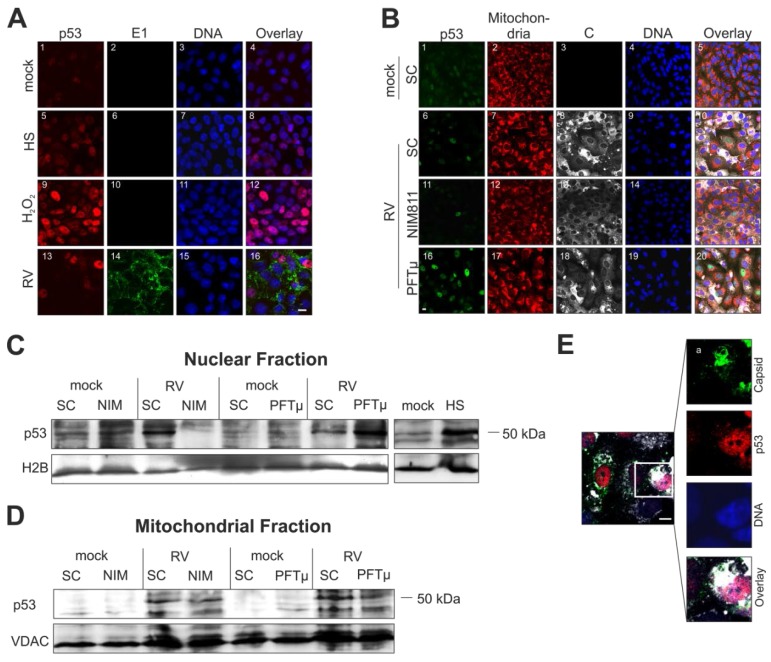
Impact of RV infection on intracellular distribution of p53. (**A**), (**B**), (**E**) Nuclear DNA is shown in blue. (**A**) Immunofluorescence analysis of mock- and RV-infected Vero cells with anti-p53 (shown in red) and anti-E1 (shown in green) at 3 dpi. Pink colour indicates overlap areas. As a positive control, heat shock (2 h at 43 °C and 24 h at 37 °C) and overnight incubation with 0.003% H_2_O_2_ was included; (**B**) Three days after infection with RV in the presence of pharmacological inhibitors NIM811 (2 μM) at 2 hpi and PFTμ (12.5 μM) at 24 hpi) the expression of p53 was compared to the SC through immunofluorescence analysis with anti-p53 antibody (shown in green) and anti-C protein antibody (shown in white). As a counterstain, mitochondria were labelled with MitoTracker Red CMXRos (shown in red) before fixation. Western blot analysis of (**C**) a nuclear and (**D**) a mitochondrial fraction obtained from RV- and mock-infected cells at 3 dpi. Conditions applied are indicated. As a loading control, anti-histone 2B (H2B) and anti-VDAC antibody were applied. HS, heat shock condition; (**E**) Immunofluorescence analysis of RV-infected Vero cells at 3 dpi, the inset depicts p53 localization (shown in red) outside the nucleus, close to areas of C protein localization (shown in green) and mitochondria (shown in white, stained with MitoTracker DeepRed). SC, solvent control; scale bar, 10 μm.

The cellular distribution of p53 was assessed in RV-infected Vero cells in the absence as well as in the presence of pharmacological inhibitors. Upon heat shock (2 h at 43 °C, followed by 24 h at 37 °C) and after overnight incubation with 0.003% H_2_O_2_, p53 nuclear localization was remarkably increased (Figure 5A, squares (8) and (12)).

Upon infection with RV, only some infected Vero cells show at 3 dpi a high p53 signal within the nucleus (Figure 5A(13)). While application of the respective inhibitors had no noticeable effect on mitochondrial morphology, differential effects on p53 distribution were observed (Figure 5B).

In comparison to the SC, application of PFTμ induced a massive p53 nuclear localization in RV-infected Vero cells at 3 dpi. The results for the SC-treated mock cell population are representative for the application of NIM811 and PFTμ. Western blot analysis of nuclear fractions confirmed the increased nuclear localization of p53 after application of PFTμ (Figure 5C). Additionally, and in contrast to the mock-infected population, Western blot analysis of mitochondria-enriched fractions from the RV-infected, SC-treated Vero cell population showed an increased band intensity for p53 which was reduced after application of PFTμ (Figure 5D). Application of NIM811 had no detectable effect on the level of p53 within the mitochondria-enriched fraction. Subsequently immunofluorescence analysis of the intracellular distribution of p53 and the mitochondria-associated viral C protein revealed punctate staining of p53 outside of the nucleus, close to C protein-positive areas (Figure 5E). These areas overlap with an area of clustered mitochondria typical for RV-infected Vero cells, which have a highly intense MitoTracker DeepRed CMX Ros staining (Figure 5E, shown in white). In summary, RV induces mitochondrial and nuclear translocation of p53. Whereas the former decreased after application of 12.5 μM PFTμ, the latter increased.

### 2.6. Rubella Virus Induces Nuclear Translocation of Cyclophilin 40

**Figure 6:**
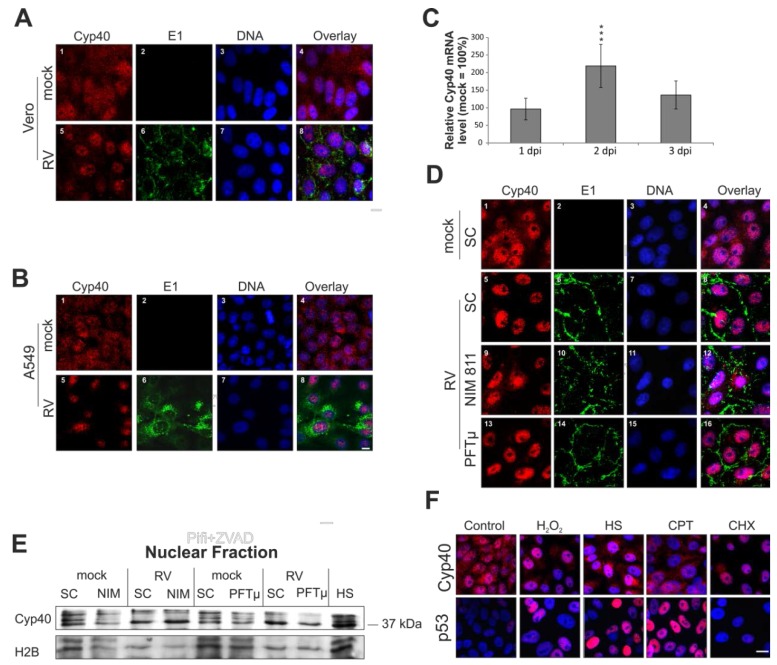
Impact of RV infection on intracellular distribution of cyclophilin 40. (**A**), (**B**), (**D**) and (**F**) Immunofluorescence analysis was performed with anti-Cyp40 (shown in red) antibody to assess intracellular distribution of Cyp40. RV infection was visualized with anti-E1 antibody (shown in green). Nuclear DNA is shown in blue. Pink colour indicates overlap areas. After fixation with 2% (*w*/*v*) paraformaldehyde, cells were permeabilized with methanol. Intracellular distribution of Cyp40 in mock- and RV-infected (**A**) Vero cells and (**B**) A549 cells at 3 dpi; (**C**) Time course analysis of the mRNA expression level of Cyp40. RNA was extracted at 1, 2, and 3 dpi from RV- and mock-infected Vero cells and subjected to analysis by quantitative real-time PCR; (**D**) Three days after infection with RV in the presence of pharmacological inhibitors (NIM811 (2 μM) at 2 hpi and PFTμ (12.5 μM) at 24 hpi) the intracellular distribution of Cyp40 was compared to the SC; (**E**) Western blot analysis of nuclear fractions obtained from mock- and RV-infected Vero cells. SC is shown in comparison to the incubation with pharmacological inhibitors NIM811 (2 μM) at 2 hpi and PFTμ (12.5 μM) at 24 hpi. Anti-H2B antibody was used as a loading control; (**F**) Intracellular distribution of p53 and Cyp40 after application of various stress stimuli, including application of heat stress (2 h at 43 °C and 24 h at 37 °C), overnight incubation with 0.003% hydrogen peroxide (H_2_O_2_), 100 μM camptothecin (CPT) and 25 μg/mL cycloheximide (CHX). SC, solvent control; scale bar, 10 μm. * *p* < 0.05, ** *p* < 0.01, *** *p* < 0.001, **** *p* < 0.0001.

The 40 kDa Cyp40 protein is involved in the cellular stress response. Figure 6A(1) shows the punctate distribution of Cyp40 within the cytoplasm of the untreated Vero population. A portion of the Cyp40 pool is also found in the nucleus. The situation in RV-infected Vero cells (3 dpi) is in strict contrast to this. Figure 6A(5) reflects a predominant localization of Cyp40 in the nucleus after RV infection. Comparable to Vero cells, a shift of Cyp40 to the nucleus is detectable at 3 dpi in RV-infected A549 cells (Figure 6B).

Thereafter, the mRNA expression level of Cyp40 was determined to verify whether this massive shift of Cyp40 to the nucleus was also associated with an increase in Cyp40 protein synthesis. Figure 6C indicates that the mRNA expression level of Cyp40 at 1 (96% ± 31%) and 3 dpi (136% ± 40%) in RV-infected Vero cells was comparable to the mock-infected population (set as 100%). However, at 2 dpi an increase in the mRNA expression level of Cyp40 was detectable (219% ± 61%).

As a next step, the effect of NIM811 (2 μM at 2 hpi) and PFTμ (12.5 μM at 24 hpi) on Cyp40 distribution was investigated. Neither the application of NIM811 nor PFTμ had a noticeable influence on Cyp40 distribution in mock-infected Vero cells, shown in Figure 6D which is representative of the SC-treated mock population. Under RV infection, Cyp40 distribution after application of PFTμ is comparable to the SC-treated population. NIM811 appears to have a minor, but negligible effect on Cyp40 distribution. Western blot analysis of nuclear fractions (Figure 6E) confirmed results obtained by the immunofluorescence analysis (Figure 6D).

Taking the influence of RV on p53 intracellular distribution into account, different stress stimuli were applied and subjected to immunofluorescence analysis, such that it could be determined whether a co-activation mechanism between these two proteins exists. Figure 6F shows that both heat shock and incubation with 0.003% hydrogen peroxide induced a shift of Cyp40 and p53 to the nucleus. The application of both camptothecin (CPT) and cycloheximide (CHX) reflects that nuclear localization of p53 and Cyp40 is not necessarily an interdependent event. CPT induces a significant portion of p53 in the nucleus, but Cyp40 remains unchanged. The application of CHX results in the opposite situation; Cyp40 is completely redistributed to the nucleus while p53 is left unaffected.

**Figure 7:**
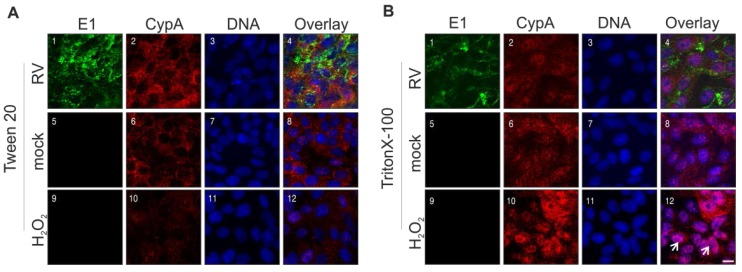
Impact of RV infection on intracellular distribution of cyclophilin A. Immunofluorescence analysis was performed with anti-CypA antibody (shown in red) and anti-E1 antibody (shown in green) for RV- and mock-infected Vero cells (3 dpi). Nuclear DNA is shown in blue. Pink colour indicates overlap areas. As a control, overnight incubation with 0.003% hydrogen peroxide (H_2_O_2_) was included. After fixation with 2% (*w*/*v*) paraformealdehyde, cells were permeabilized with 0.1% TritonX-100 (**A**) or with 0.3% Tween 20 (**B**). Arrows point to representative cells with a clear shift of CypA to the nucleus. Scale bar, 10 μm.

Finally, the intracellular distribution of cyclophilin A (CypA), which is involved in apoptotic processes and acts as a host factor for several viruses [24], was investigated. Immunofluorescence analysis was performed after permeabilization with two different detergents. Triton X-100 partially dissolves the nuclear membrane, while Tween 20 enables staining of mainly cytoplasmic proteins. Figure 7A highlights that after permeabilization with Tween 20, hardly any differences between mock- and RV-infected Vero cells and Vero cells after overnight incubation with 0.003% hydrogen peroxide are noticeable. This is in contrast to permeabilization with Triton X-100, which shows a clear shift of CypA from a cytoplasmic to a nuclear localization (Figure 7B(12)). However, as compared to the mock-infected control, only a slight shift of CypA to the nucleus can be speculated after infection with RV (Figure 7B(4,8)). In conclusion, during RV infection, nuclear translocation of Cyp40, but not of CypA, is detectable.

### 2.7. Rubella Virus Reveals Strain-Specific Effects in Its Mode of Apoptosis Induction

**Figure 8:**
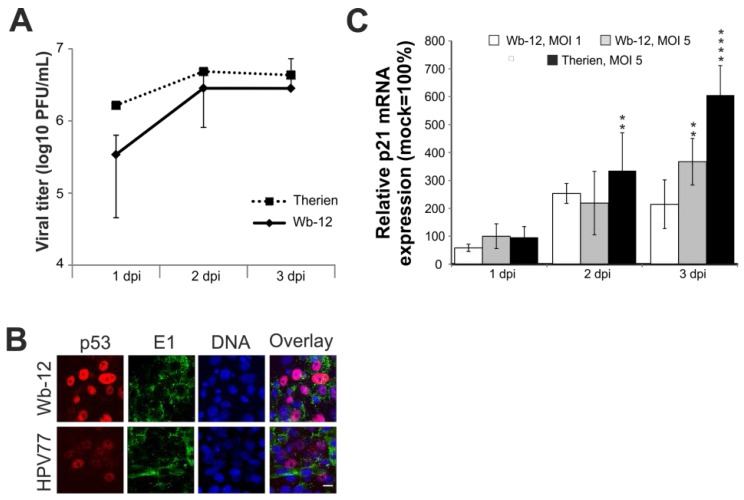
The course of infection of the clinical isolate RVi/Wuerzburg.DEU/47.11 (Wb-12) as compared to the cell culture-adapted strains Therien and HPV77. (**A**) Determination of Wb-12 viral titer over time of infection (1 to 3 dpi) by plaque assay in comparison to Therien; (**B**) Immunofluorescence analysis was performed with anti-p53 antibody to assess intracellular distribution of p53 (shown in red). RV infection was visualized with anti-E1 antibody (shown in green). Nuclear DNA is shown in blue. Pink colour indicates overlap areas; (**C**) Alteration of the mRNA expression level of p21 after infection with RV strains, Therien and Wb-12. The mRNA expression level was determined at 1, 2 and 3 dpi relative to the corresponding mock sample (set at 100%). Scale bar, 20 μm. * *p* < 0.05, ** *p* < 0.01, *** *p* < 0.001, **** *p* < 0.0001

Comparable to the vaccine strain HPV77, the selection of the Therien strain for its high replication rate in Vero cells was the result of a cell-culture adaptation process. CPE induction by a low-passaged clinical isolate, represented by the strain Wuerzburg-12 (Wb-12, RVi/Wuerzburg.DEU/47.11) was therefore addressed. The viral titer generated during Wb-12 infection is comparable to the one observed for Therien (representative graph in Figure 8A and [25]). Additionally, CPE development by Wb-12 was comparable to Therien in terms of the onset of cell detachment. However, the number of floaters detected at 3 dpi per well of a six-well plate was slightly reduced for Wb-12 ((2.25 ± 2.29) × 10^5^ floaters per mL, *n* = 3) as compared to Therien (9.28 ± 2.25) × 10^5^. Under Therien infection, only a slight shift of p53 to the nucleus was detectable (Figure 5A), which is comparable to the infection by HPV77, but more profound after infection with Wb-12 (Figure 8B).

Taking this high level of nuclear translocation of p53 into account, the mRNA expression level of p21^Waf1/Cip1^ (p21) was determined, which is regulated by the transcriptional activity of p53. Despite the intensive nuclear localization of p53 under Wb-12 infection, the induction of p21 mRNA expression is lower than the one observed for Therien (Figure 8C). Results presented point to nuclear localization of p53 as a distinguishing feature between RV cell-culture-adapted strains and clinical isolates.

## 3. Discussion

**Figure 9:**
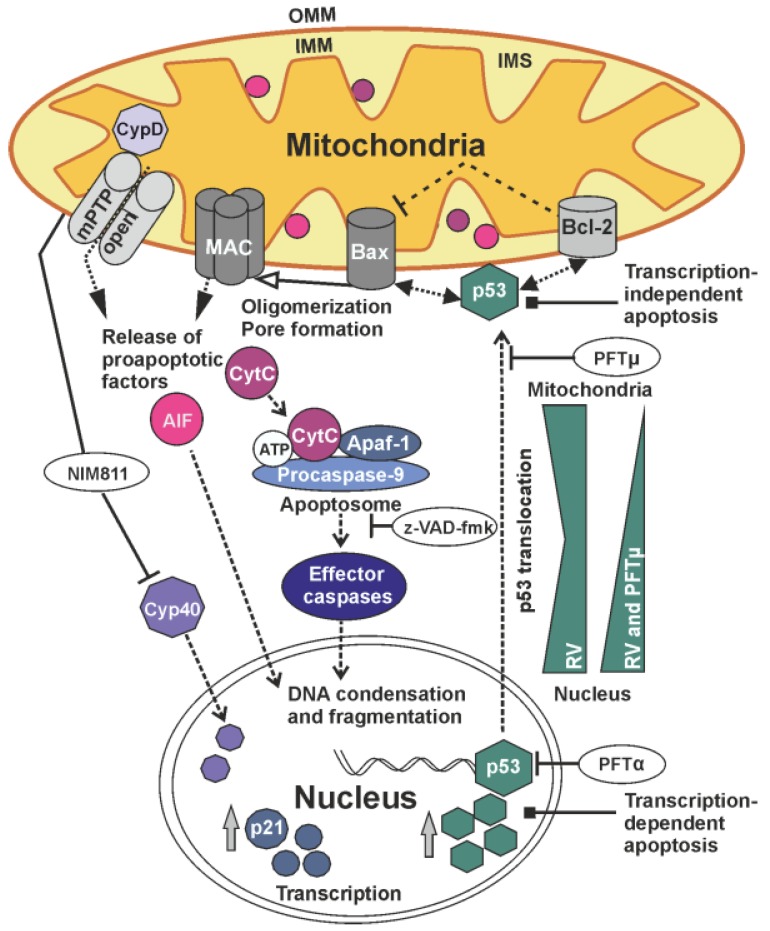
Proposed model for the involvement of mitochondria- and p53-associated pathways during RV-induced cell death. The transcription-independent mitochondrial p53 function can be blocked by PFTμ. p53 can interact with the pro-apoptotic protein Bax to induce its oligomerization and formation of the mitochondrial apoptosis-induced channel (MAC). The release of pro-apoptotic CytC promotes the formation of the so-called apoptosome consisting of apoptotic protease-activating factor (Apaf-1), pro-caspase 9 and CytC. Effector caspases can be inhibited by the pan-caspase-inhibitor z-VAD-fmk. Application of NIM811 can inhibit cyclophilins such as CypD and Cyp40 as examined in this study. Further explanations are provided in the manuscript. IMS, mitochondrial intermembrane space.

Mitochondria are crucial in the execution of programmed cell death and therefore an important line of defense against various insults such as pathogenic infections. Hence some proteins of important human pathogenic viruses do even localize within mitochondria to interfere with apoptosis [26]. Another important cellular factor for viral countermeasures against cellular defense mechanisms is the tumor-suppressor protein p53 as an important orchestrator of those intertwined and interdependent mitochondrial functions [27]. The data presented in this paper indicate that RV-induced apoptosis as well as its cell-culture adaptation involve a balance between nuclear and extranuclear functions of p53. Additionally, RV induces release of pro-apoptotic proteins from mitochondria and nuclear translocation of Cyp40. The presented data are summarized in Figure 9 as a proposed model for the mitochondria- and p53-associated pathways during RV-induced apoptosis.

Through the application of PFTμ, this paper indicates that p53-based pathways are involved in programmed cell death mechanisms induced by RV infection. In comparison to the Therien and HPV77 strains, the clinical isolate Wb-12 induced a stronger signal of nuclear p53. This suggests that during cell-culture adaptation RV strains have evolved mechanisms to counteract or to avoid nuclear localization of p53. The application of PFTμ appears to support the previously published anti-apoptotic activity of Therien, which is also reflected by the association of its C protein with Bax [7]. The present paper ties in with this reported involvement of a mitochondrial signaling pathway in RV-infected A549 cells and subsequently viral strategies to counteract cellular death mechanisms. Additionally, application of PFTμ to RV-infected Vero cells increases p53 nuclear expression during RV infection, highlighting that blocking one branch of p53 activity favors the other. However, Therien appears not to be affected by the PFTμ-associated increase in nuclear p53 localization, as PFTμ reduces CPE.

Cyclophilins act as important co-factors for a number of viruses [28] and are thus a promising target for antiviral countermeasures [29]. Nevertheless, there are also cases in which cyclophilins restrict viral replication [24]. No cellular functions that are vital for RV replication were affected by the application of NIM811 as reflected by the maintained virus production rate. It is important to note that RV specifically affects the expression pattern of the ubiquitous protein Cyp40 that acts as co-chaperone of the Hsp90/Hsp70 complex and guides its interaction with other proteins, including those involved in antiviral host response [30]. Cyp40 is occasionally referred to as CypD, which contributes to some unresolved issues in its physiological functions. Its participation in protein maturation pathways [17], especially its nuclear localization after stress induction by UVA treatment [31], heat stress [32], or RV infection as shown here, certainly warrants further exploration and points to a multitude of physiological functions. Especially heat stress parallels some of the effects exerted by RV on its host cell, including Cyp40 nuclear redistribution and an increase in the Cyp40 mRNA level [32]. Noteworthy in this context is that Hsp90-binding cyclophilins such as Cyp40 are involved in protein trafficking to the nucleus, including p53 [33].

The release of apoptosis-promoting factors upon mitochondrial membrane permeabilization is crucial for the execution of the intrinsic mitochondrial apoptotic pathway. Data presented in this paper highlight mPTP opening and mitochondrial-nuclear translocation of AIF in a minor portion of cells under RV infection. Being released from mitochondria, AIF is a potent apoptosis-promoting factor [34]. Its nuclear translocation is followed by chromatinolysis and DNA fragmentation. However, the contribution of AIF and cyclophilins to RV-associated cell death appears to be negligible as indicated by the minor effect exerted by NIM811 on RV floater population or on selected markers for apoptosis. The observed release of CytC could help to explain the reported reduction of mitochondrial respiratory chain complex IV activity during RV infection [12].

The regulation of mPTP composition and function by CypD is still under an ongoing discussion [21,22,23]. Overexpression of wild-type (WT) CypD and its PPIase mutants suggests a PPIase-independent involvement of CypD in RV-associated cell death. Hence, in RV-infected Vero cells, its apoptosis regulation and protein-folding functions appear to be separated. This notion also applies to the interaction of CypD with some of its protein partners, including hexokinase II [35] and the adenine nucleotide translocase (ANT) [36]. Additionally, a modulation of mPTP independent from its enzymatic activity was reported [37]. It was just published that mPTP is a regulatory component of vascular function [38], which should be viewed in the light of the perturbance of angiogenic processes seen with patients suffering from congenital rubella syndrome (CRS) [39].

This study highlights the importance of not only considering cell specificity in the course of virus-induced apoptosis, but also virus strain specificity. These observations are strengthened by studies on viral strain-specific cytopathogenicity of human respiratory syncytial virus, cytomegalovirus and coronavirus [40,41,42]. However, the use of just one cell line for most of the results shown in this study limits the conclusions that can be drawn for RV-induced apoptosis. In future studies, the observed apoptotic effects induced by RV strains in Vero cells, which are of simian origin, need to be further addressed in relevant human cell lines such as fetal fibroblasts and the A549 cell line. Nevertheless, we have provided some verification in our study that the findings reported are not restricted to Vero cells.

There is a well-defined balance between cell survival and cell-death induction, and consequently the cellular responses to a viral infection are interconnected with the resulting viral countermeasures. Already minor changes can evoke alterations of cellular antiviral activities, as shown in this paper by the increase in p53 nuclear expression after inhibiting mitochondrial shuttling of p53 with PFTμ. CPE induction after RV infection is only observed after infection of susceptible cell lines such as Vero and RK13 [2]. It is especially noteworthy that RV-induced apoptosis is detectable in human primary differentiated, nonproliferative cells, but not in proliferative fetal cells [43]. This strengthens the cellular context dependency of RV-associated apoptotic processes, which might be due to differences in cellular signaling pathways or to differences in the anti-viral response provoked by RV in these cell lines. The identification of cellular factors specific for RV-induced cell death as outlined in this study will help to address and delineate this cell line-dependency as identified factors can be specifically examined in their cellular context. Clinical isolates can be regarded as a tool for identification of viral and cellular components that contribute to RV cell-culture adaptation. Future experiments will be aimed at elucidating the interplay of RV strains with nuclear and extranuclear p53 pools and how a virus coordinates cellular functions to its own advantage.

## 4. Materials and Methods

### 4.1. Antibodies and Pharmacological Reagents

Mouse antibody against AIF and rabbit antibodies against p53, H2B, and Cyp40, respectively, were purchased from Santa Cruz Biotechnology; mouse antibody against CytC and rabbit antibodies against VDAC and CypA were from Abcam. Monoclonal mouse anti-E1 antibody was from Viral Antigens, and mouse anti-alpha tubulin was obtained from Sigma Aldrich (St. Louis, MO, USA). Secondary antibodies for immunofluorescence and Western blot analysis were from Dianova. The pan caspase inhibitor z-VAD-fmk was from PeptaNova (Sandhausen, Germany); PFTμ and α, staurosporine and camptothecin were purchased from Santa Cruz Biotechnology (Dallas, TX, USA). NIM811 was generously provided by Novartis (Basel, Switzerland). All other reagents were from Sigma Aldrich. Stock solutions were prepared in dimethyl sulfoxide (DMSO), stored at −20 °C and diluted in cell culture medium to their respective final concentrations directly before use. Final concentration of DMSO, which was employed as vehicle (solvent) control, never exceeded 0.1%.

### 4.2. Vero Cell Cultivation, Drug Treatment and Virus Strains

p53- and cyclophilin-regulated pathways are generally altered in cancer cells, hence experiments were mainly performed with Vero, a standard and non-tumor-derived cell line for RV cultivation. Additionally, CPE by RV is very profound in this cell line [18]. Several RV-associated cell culture aberrations were characterized in this cell line, including the analysis of the p53 dependency [5]. Vero cells are negative for the type I interferon response [44], which allows the separation of RV-induced effects on mitochondria-based pathways from effects that are triggered through generation of type I interferons [45]. Vero and A549 cells were maintained in DMEM, supplemented with fetal bovine serum (10%) and antibiotics at 37 °C in a humidified incubator with 5% CO_2_ atmosphere. Medium for A549 cells was supplemented with 1 mM HEPES. After overnight cultivation, cells were mock- and RV-infected and treated with pharmacological compounds or vehicle (solvent control) only. Medium was removed and effectors were added in fresh medium at indicated time points. Cells were incubated in the presence of the respective effector until sample processing. The laboratory-adapted F-Therien strain was applied to Vero cells at an multiplicity of infection (MOI) of 5 unless otherwise indicated. Besides the Therien strain, the vaccine strain HPV77 and the clinical isolate Wb-12, a kind gift of B. Weißbrich (University of Wuerzburg, Wuerzburg, Germany), were used. The Wb-12 strain was passaged seven times on Vero cells after its initial isolation. An MOI of 5 was chosen to ensure an optimal infection rate resulting in a high level of CPE induction. The application dose of the pharmacological compounds was assessed by classical trypan blue exclusion test and was within the published range.

### 4.3. Plasmids and Transient Transfection

CypD-overexpression plasmids pcDNA3.1-HA-cyclophilin D/WT (pcCypD), pcDNA3.1-HA-cyclophilin D/R97A (pcR97A), and pcDNA3.1-HA-cyclophilin D/H168Q (pcH168Q) were provided by M. Kawatani (RIKEN Advanced Science Institute, Saitama, Japan). Plasmid pAIF-TagRFP based on pTagRFP-N1 (Evrogene) was provided by Dr M. Varecha (Centre for Biomedical Image Analysis, Faculty of Informatics, Masaryk University, Brno, Czech Republic). At 2 hpi Vero cells were transiently transfected with *Trans*IT®-LT1 (Mirus Bio LLC, Madison, WI, USA) following manufacturer’s instructions.

### 4.4. Characterization of Floaters: DNA Fragmentation Assay

As a marker for cellular alterations caused by RV, detached floater cells were collected through centrifugation of cell culture supernatants obtained from the respective well of a six-well plate. For the DNA fragmentation assay, floater cells were collected from 175 cm^2^ flasks. As a positive control, 6 × 10^6^ adherent Vero cells were incubated with 5 μM staurosporine for 90 min at 37 °C. Afterwards, medium change cells were incubated for an additional 3 h and thereafter subjected to trypsinization. After washing of the detached control cells and the floater population (at 1 × 10^6^ cells per sample) with PBS, the samples were treated with lysis buffer (100 mM Tris pH 8.0, 20 mM EDTA, 0.8% SDS) for 20 min at room temperature. Thereafter, cells were incubated with 10 μL RNAse A solution (500 U/mL) for 2 h at 37 °C and afterwards digested with 10 μL proteinase K (20 mg/mL) overnight at 50 °C. After the addition of NaCl and PEG-800 at a final concentration of 1 M and 2.5%, respectively, samples were incubated on ice for 10 min and centrifuged at 4 °C for 10 min at 16,000× *g*. The resulting supernatant was collected before sodium acetate and ethanol were added at 1/10 and twofold volumes, respectively. Incubation at −80 °C for 1 h resulted in DNA precipitation. After centrifugation at 16,000× g at 4 °C the supernatant was discarded and the remaining pellet was washed with ice-cold 70% (*v*/*v*) ethanol and dissolved in 40 μL ddH2O. The DNA was analyzed by 1.5% standard agarose gel electrophoresis at 60 V for 30–60 min.

### 4.5. Cell Death Assays

Induction of apoptotic cell death was monitored by the binding of annexin V-EGFP (Annexin V-EGFP Apoptosis Detection Kit from GenScript (Piscataway, NJ, USA) according to the manufacturer’s instructions. Viable cells are not permeable for PI (5 μM), while induction of apoptosis leads to binding of annexin V due to exposure of phosphatidylserine. Activity of caspase 3 and 7 was determined using the CellEvent caspase-3/7 ReadyProbes® reagent (Thermo Fisher Scientific, Braunschweig, Gemany) according to manufacturer’s instructions. Briefly, Caspase-3/7 activity was determined by microscopic analysis of the fluorescent events after cleavage of the non-fluorescent DEVD peptide. As a DNA counterstain, cells were stained with Hoechst bisbenzimide 33342 (Thermo Fisher Scientific, Braunschweig, Gemany), (2 μM). Samples were analyzed by fluorescence microscopy using a FITC/TRITC filter.

### 4.6. Calcein Release Assay for Assessment of Mitochondrial Permeability Transition Pore (mPTP) Opening

mPTP opening was assessed with the MitoProbe transition pore opening kit (Thermo Fisher Scientific Life Technologies). Mitochondrial calcein is lost after the opening of the mPTP. Hence, the mPTP opening was assessed through evaluation of the mitochondrial fluorescence signal of calcein after the quenching of its cytosolic portion with CoCl_2_. Briefly, medium was replaced with warm Hank’s balanced salt solution containing calcium (HBSS/Ca) and loaded with calcein-acetoxymethyl ester (calcein-AM at 1.0 μM) and CoCl_2_ (1.0 mM). After an incubation of 3 h at 37 °C, cells were washed with HBSS and directly processed to fluorescence microscopic analysis. Opening of mPTP was induced through overnight incubation with H_2_O_2_ (0.003%).

### 4.7. Immunofluorescence Analysis

Cells were fixed with paraformaldehyde (2% *w*/*v*), permeabilized with methanol, 0.1% Triton X-100, 0.3% Tween 20, or digitonin as indicated. Unspecific binding was blocked through incubation with 5% goat serum for 1 h at 37 °C. After intensive washing incubation with primary antibody (the anti-E1 antibody was used at a 1:200 dilution in PBS, anti-CytC at 1:250, anti-CypA and anti-alpha tubulin at 1:500, all other antibodies at 1:100) was performed for 1 h at 37 °C. Secondary antibodies (DyLight 488- or Cy3-labelled, Dianova GmbH (Hamburg, Germany) were used at a 1:100 dilution and incubated at 37 °C for 45 min, thereafter as nuclear counterstain Hoechst bisbenzamide 33285 (5 μg/mL, Thermo Fisher Scientific) was applied. As a mitochondrial counterstain, MitoTracker Red CMXRos was applied according to the instructions of the MitoProbe transition pore opening kit. Samples were mounted in Entellan and images were taken with the Zeiss510 confocal microscope (Zeiss, Oberkochen, Germany) and processed using CorelDRAW X7 (Corel Corporation, Ottawa, Ontario, Canada) with minimal alterations to brightness and contrast.

### 4.8. Cell Fractionation and Western Blot Analysis

Nuclear fractionation was performed by incubation of confluent cells grown in a 60 mm dish (initially 1 × 10^6^ cells were plated) in 500 μL buffer A (10 mM HEPES, 1.5 mM MgCl_2_, 10 mM KCl, 0.5 mM DTT, 0.05% NP-40, pH 7.9) supplemented with 1 mM PMSF and protease inhibitor mix for 15 min on ice under shaking conditions. Lysed cells were centrifuged at 3000 rpm and 4 °C for 10 min. The pellet was resuspended in 94 μL buffer B (5 mM HEPES, 1.5 mM MgCl_2_, 0.2 mM EDTA, 0.5 mM DTT, 26% (*v*/*v*) glycerol pH 7.9) supplemented with 1 mM PMSF and protease inhibitor mix. Each sample was charged with 6 μL 5 M NaCl and incubated under shaking conditions for 2 h at 4 °C. Finally, samples were centrifuged at 13,000 rpm for 20 min at 4 °C and the supernatant was collected as nuclear fraction. Mitochondria-enriched fractions were obtained by differential centrifugation as published [11]. Mitochondrial (30 to 120 μg/lane) and nuclear (20 μg/lane) fractions were subjected to SDS polyacrylamide gel electrophoresis and transferred to a PVDF membrane for detection with primary (anti-VDAC antibody was diluted 1:2000, all other antibodies 1:200) and HRP-conjugated secondary antibodies as described [11]. Equivalent protein loading in the mitochondrial and nuclear fraction was monitored with VDAC and histone 2B (H2B), respectively.

### 4.9. RNA Preparation and Real-Time Quantitative PCR

Total RNA was extracted and reverse transcribed and analyzed by quantitative real-time PCR as published [25]. Reactions were performed in a total of 20 μL containing 10 μL GoTaq® qPCR (2×) Master Mix (Promega, Mannheim, Germany) and 1 μg BSA. According to reference sequence NM_005038 primer sequences were selected for Cyp40, 5′ tgaaaggtgaaaaacctgctaaa 3′ for the sense primer CyP40.s, and 5′ cagagccatcttttgggaata 3′ for the antisense primer CyP40.as. An amplicon of 96 bp was generated. The p21 primer sequences are as published [25].

### 4.10. Statistics

All data in the diagrams are expressed as means ± standard errors (SE) of at least three individual experiments. One-way ANOVA followed by Tukey’s post hoc analysis was applied to determine statistical significance using Graph Pad Prism software (GraphPad Software, Inc., La Jolla, CA, USA). Asterisks indicate level of significance (* *p* < 0.05, ** *p* < 0.01, *** *p* < 0.001, **** *p* < 0.0001).

## 5. Conclusions

There are conflicting reports on the mode of rubella virus (RV)-associated cell death and on possible differences between viral strains. Through application of selected pharmacological inhibitors, the present study highlights the contribution of mitochondria- and p53-centered apoptotic pathways to cytopathic effect induction by RV. Additionally, the relevance of the so far not well-characterized Cyp40 protein in the cellular stress response was emphasized. Our present work also shows that p53 may contribute to differences among RV cell culture-adapted strains and a low-passaged clinical isolate.
